# Immune checkpoint blockade in cancer: current insights and future horizons

**DOI:** 10.1007/s12672-025-04361-7

**Published:** 2026-01-02

**Authors:** Harman Saman, Karama Makni-Maalej, Dina M. Abo El-Ella, Maryam Yousef Al-Tamimi, Mohannad N. AbuHaweeleh, Nada Abuhayeh, Mohamed A. Ismail, Kirti S. Prabhu, Queenie Fernandes, Thameema Jabi, Rayan Elhussein, Varghese Philipose Inchakalody, Maysaloun Merhi, Said Dermime, Shahab Uddin

**Affiliations:** 1 Internal Medicine, Respiratory Medicine, Medical Oncology, Hamad Medical Corporation, Doha, Qatar; 2 Barts Cancer Institute, Queen Mary University of London, London, EC1M 5PZ UK; 3 Translational Cancer Research Facility, National Center for Cancer Care and Research, Hamad Medical Corporation, Doha, Qatar; 4 College of Medicine, Qatar University, Doha, Qatar; 5 College of Pharmacy, Qatar University, Doha, Qatar; 6 Hamad Medical Corporation, Doha, Qatar; 7 Translational Research Institute, Academic Health System, Hamad Medical Corporation, P.O. Box 3050, Doha, Qatar; 8 Research Department, Weill Cornell Medicine, Doha, Qatar; 9 College of Health Sciences, Qatar University, Doha, Qatar; 10 College of Health and Life Sciences, Hamad Bin Khalifa University, Doha, Qatar; 11 Dermatology Institute, Academic Health System, Hamad Medical Corporation, Doha, Qatar; 12 Laboratory of Animal Research Center, Qatar University, Doha, Qatar

**Keywords:** Immune checkpoint inhibitors, Tumour microenvironment, T cells exhaustion, Cancer immune evasion, Cancer treatment, Biomarkers, Immune-related adverse events, Immune checkpoint targets

## Abstract

Immunotherapy represents a paradigm shift in oncology, rooted in a century of evolving scientific understanding and clinical application. From the pioneering use of Coley’s toxins in the late nineteenth century to the introduction of cytokine-based interventions, the trajectory of immunotherapeutic approaches has paralleled advancements in immunology and molecular biology. This review comprehensively examines the historical development and progressive refinement of immunotherapy for cancer, charting the transition from non-specific immune stimulation to targeted immune modulation. Central to this discussion are the sophisticated mechanisms by which tumour cells evade immune detection and destruction. These include downregulation of antigen presentation machinery, secretion of immunosuppressive cytokines, recruitment of regulatory T cells and myeloid-derived suppressor cells, and exploitation of immune checkpoint pathways, particularly CTLA-4 and PD-1/PD-L1 axes. The advent of immune checkpoint inhibitors has yielded durable clinical responses in diverse malignancies, substantiating their role as foundational agents in cancer therapy. Nonetheless, both primary and acquired resistance to immune checkpoint inhibition remain significant clinical obstacles. Resistance mechanisms are multifactorial, involving tumour-intrinsic genetic alterations, modulation of the tumour microenvironment, and adaptive changes in immune cell phenotypes. Contemporary research endeavors are directed at overcoming these barriers, including the optimization of combinatorial regimens, development of next-generation checkpoint modulators, tumour-specific vaccines, and the integration of adoptive cell therapies. Future directions in cancer immunotherapy are poised to leverage advances in systems biology, genomics, and single-cell technologies to individualize interventions and enhance therapeutic efficacy. Ultimately, a comprehensive delineation of tumour-immune interactions will underpin the next generation of rational, effective, and durable cancer immunotherapies.

## Introduction

The emergence of immunotherapy as a viable modality in cancer treatment marks a significant milestone in the history of oncology. Early attempts to harness the immune system against malignancy began in the late nineteenth century, most notably with William Coley’s use of heat-killed bacterial toxins, an approach based on observations that infections sometimes coincided with tumour regression [1]. Although rudimentary by contemporary standards, these pioneering experiments laid the conceptual groundwork for later scientific inquiry into the interplay between host immunity and tumour progression. Over several decades, growing evidence from immunological research suggested that the immune system could recognize and occasionally eliminate cancer cells. However, the complexity and redundancy of immune regulatory mechanisms posed considerable challenges for translating these insights into reliable clinical therapies [2, 3].

Advancements in molecular and cellular immunology throughout the late 20th and early twenty-first centuries transformed the landscape of cancer immunotherapy. The identification and characterization of immune checkpoints, such as cytotoxic T-lymphocyte-associated antigen 4 (CTLA-4) and programmed cell death-1 (PD-1), provided crucial new targets for therapeutic intervention, catalysing the development of immune checkpoint inhibitors (ICIs). These agents, by blocking inhibitory pathways exploited by tumour cells, have demonstrated unprecedented efficacy in a range of malignancies, often resulting in durable responses not observed with conventional cytotoxic therapies. Concurrently, the evolution of adoptive cell transfer, cancer vaccines, and cytokine-based therapies further broadened the therapeutic arsenal, underscoring the versatility and potential of immunomodulation in oncology. As the field continues to evolve, immunotherapy is increasingly integrated into synergistic, multimodal treatment strategies, heralding an era where utilising the immune system is a central pillar of cancer care [4, 5].

The tumour microenvironment (TME) is an intricate and dynamic system comprising not only malignant cells but a heterogeneous population of stromal fibroblasts, endothelial cells, pericytes, immune cells, and the surrounding extracellular matrix (ECM) [6, 7]. This ecosystem is controlled through tightly regulated cellular and molecular interactions, supporting tumour progression and dissemination [8, 9]. The molecular interactions between tumour cells and stromal components help create an environment that supports tumour growth by encouraging new blood vessel formation, increasing cell multiplication, and enabling cells to resist death. As a result, the tumour microenvironment is considered a key factor in cancer development. The TME is therefore recognized as a critical determinant of cancer behavior and therapeutic response, rather than a passive bystander [6, 10, 11].

A prominent facet of the TME is its profound capacity to suppress anti-tumour immune responses. Tumour cells release a broad array of immunomodulatory factors, including cytokines such as transforming growth factor-beta (TGF-β) and interleukin-10 (IL-10), which actively inhibit the recruitment, activation, and cytotoxic function of effector immune cells [10, 12]. Simultaneously, the TME supports the accumulation and expansion of immune-regulatory cell populations such as T-regs, myeloid-derived suppressor cells (MDSCs), and tumour-associated macrophages (TAMs) with an M2 phenotype. These cells secrete additional immunosuppressive mediators, alter cytokine profiles, and metabolically reprogram the microenvironment, further compounding immune evasion [13, 14].

One of the definitive immunosuppressive mechanisms employed within the TME involves the upregulation of immune checkpoint pathway components, most notably programmed cell death-ligand 1 (PD-L1) on tumour and stromal cells [15]. Upon interaction with its cognate receptor PD-1 on activated T cells, PD-L1 transmits inhibitory signals that attenuate T cell receptor (TCR) signaling, resulting in T cell exhaustion and functional anergy [6]. This weakening of cytotoxic lymphocyte activity effectively prevents the immune-mediated clearance of tumour cells, facilitating continued tumour progression even in the context of an inflamed microenvironment. Similar checkpoint pathways, including CTLA-4, lymphocyte-activation gene 3 (LAG-3), and T-cell immunoglobulin and mucin-domain containing-3 (TIM-3), are often co-opted by tumour and stromal components, collectively establishing a multilayered barrier to effective anti-tumour immunity [14, 16, 17].

Beyond immune modulation, the physical and metabolic characteristics of the TME also restrict immune cell efficacy. The ECM can function as a physical barricade to immune cell infiltration, while abnormal tumour vasculature generates hypoxic conditions and impedes effective delivery of immune effector cells [18]. Hypoxia, acidosis, and nutrient deprivation further restrict local immune cell metabolism and function, shifting the balance toward tolerance and immune escape [19, 20]. For example, the accumulation of metabolic byproducts such as adenosine and lactic acid suppresses T cell proliferation and effector function, while concurrently fostering a milieu favourable to regulatory myeloid populations [9, 21, 22].

Given these multifaceted immunosuppressive strategies, therapeutic interventions targeting the TME are a focal point of contemporary cancer research [6, 21]. Immune checkpoint inhibitors, which block the PD-1/PD-L1 or CTLA-4 axes, have demonstrated remarkable clinical benefit across various malignancies, validating the centrality of TME-mediated immunoregulation in cancer pathogenesis [6, 11]. Combination therapies aimed at modulating cytokine networks, depleting immunosuppressive cell subsets, reprogramming the ECM, or normalizing tumour vasculature are actively being investigated to overcome resistance to immunotherapy. A detailed understanding of TME-driven immunosuppression is essential for the continued development of rational, effective cancer Immunotherapeutics [10, 11].

## Immune checkpoint inhibitors: a paradigm shift

The discovery and clinical validation of immune checkpoint pathways have revolutionized cancer therapy. Immune checkpoints are regulatory molecules (e.g., CTLA-4, PD-1) that normally prevent autoimmunity by inhibiting excessive immune activation. However, tumours exploit these checkpoints to suppress anti-tumour immunity [23].

The clinical translation of this mechanistic insight was exemplified by the U.S. Food and Drug Administration’s (FDA) 2011 approval of ipilimumab, a monoclonal antibody targeting CTLA-4, for the treatment of metastatic melanoma [24, 25]. This milestone catalyzed the development of subsequent generations of ICIs that inhibit the PD-1/PD-L1 axis [26]. Revolutionary agents such as nivolumab and pembrolizumab, both directed against PD-1, as well as PD-L1-specific inhibitors like atezolizumab and durvalumab, have since expanded the therapeutic repertoire available to clinicians. Since the first approval of iplilimuman, FDA- and European Medicines Agency (EMA)-approved a range of agents including anti-PD-1 antibodies pembrolizumab, nivolumab, cemiplimab, dostarlimab, toripalimab, sintilimab, camrelizumab and spartalizumab; and anti-PD-L1 antibodies atezolizumab, durvalumab and avelumab [27, 28]. Less frequently used ICIs include relatlimab (Opdualag) and tremelimumab (Imjudo) (both FDA approved in 2022) are used in combination with another ICI. For example Relatlimab—an anti-LAG-3 antibody approved in combination with nivolumab (Opdualag) by FDA and EMA for advanced melanoma [29, 30]. Whereas Tremelimumab an anti-CTLA-4 antibody approved in combination with durvalumab for specific indication namely unresectable hepatocellular carcinoma (HCC) [31, 32]. An important difference amongst these agents, is the structure of the Fc effector potential and pharmacokinetics, which affect tissue distribution, synergistic with other anti-cancer agents and importantly the side effects profile [33].

These immunotherapeutic drugs have demonstrated substantial efficacy across an expanding spectrum of malignancies. Their clinical utility has been established in non-small cell lung cancer, renal cell carcinoma, classical Hodgkin lymphoma, and urothelial carcinoma, among others [34–36]. Importantly, these benefits have been observed in both first line and subsequent treatment settings, underscoring the versatility of ICIs in diverse disease contexts. Notably, several ICIs have received FDA approval for various cancer indications, including ipilimumab, nivolumab, pembrolizumab, atezolizumab, and more recently tislelizumab and cosibelimab-ipdl, as outlined in Table 1. The spectrum of their activity continues to broaden as ongoing trials investigate additional tumour types and combinatorial regimens.

**Table 1:** Examples of FDA approved ICIs

Drug name	Target	Mechanism	FDA approval year	Primary indications
Ipilimumab	CTLA-4	Anti-CTLA-4 monoclonal antibody	2011	Melanoma, Mesothelioma
Pembrolizumab	PD-1	Anti PD-1 monoclonal antibody	2014	NSCLC, melanoma, multiple cancer
Nivolumab	PD-1	Anti PD-1 monoclonal antibody	2014	NSCLC, melanoma, RCC, multiple cancer
Atezolizumab	PD-L1	Anti PD-L1 monoclonal antibody	2016	NSCLC, Urothelial Cancer, Small Cell Lung Cancer, Breast Cancer
Avelumab	PD-L1	Anti PD-L1 monoclonal antibody	2017	Merkel cell carcinoma, Urothelial cancer
Durvalumab	PD-L1	Anti PD-L1 monoclonal antibody	2017	Urothelial cancer, NSCLC
Cemiplimab	PD-1	Anti PD-1 monoclonal antibody	2018	Cutaneous squamous cell carcinoma
Dostarlimab	PD-1	Anti PD-1 monoclonal antibody	2021	Endometrial cancer, MSI-H solid tumors
Retifanlimab	PD-1	Anti PD-1 monoclonal antibody	2023	Merkel cell carcinoma
Relatimab	LAG-3	Anti-LAG-3 monoclonal antibody	2022	Advanced melanoma (with nivolumab)
Cosibelimab	PD-L1	Anti PD-L1 monoclonal antibody	2024	Cutaneous squamous cell carcinoma

*NSCLC* non-small cell lung cancer, *RCC* renal cell carcinoma, *MSI-H* microsatellite instability-high

A large corpus of randomized clinical trial (RCT) data supports the superiority of ICIs with respect to key clinical endpoints. Meta-analyses have consistently revealed significant improvements in overall survival (OS) and progression-free survival (PFS) among patients treated with ICIs relative to traditional cytotoxic chemotherapies [37–40]. This paradigm shift has been particularly pronounced in metastatic melanoma, where durable, complete responses have occurred, an outcome that was previously unattainable with conventional modalities [39, 40].

## Immune checkpoint pathways

### Role of CTLA-4, PD-1, and PD-L1 in immune regulation

The immune system contains complex regulatory mechanisms known as immunological checkpoints, essential for preserving self-tolerance and regulating the strength and duration of immune responses to avert excessive inflammation or autoimmunity [41]. These checkpoints involve specific receptor-ligand interactions that can either stimulate or inhibit T-cell activity. While co-stimulatory pathways enhance immune responses, co-inhibitory pathways, such as those involving CTLA-4 and PD-1, act as crucial brakes on T-cell activation and function [42].

CTLA-4, or CD152, is a crucial inhibitory receptor mostly expressed on activated conventional T cells and consistently present on regulatory T cells (Tregs). Its expression is rapidly upregulated following TCR activation [43–45]. CTLA-4 functions mainly during the initial priming phase of T-cell activation, typically occurring in secondary lymphoid organs like lymph nodes [45]. It acts as a direct competitor to the co-stimulatory receptor CD28. Moreover, CTLA-4 and CD28 interact with the same ligands, CD80 (B7-1) and CD86 (B7-2), which are expressed on antigen-presenting cells (APCs) [45]. However, CTLA-4 binds these ligands with significantly higher affinity and avidity than CD28 [46]. CTLA-4 diminishes T-cell activation by outcompeting CD28 for B7 ligand binding, thereby restricting T-cell proliferation and decreasing the synthesis of critical cytokines such as Interleukin-2 (IL-2). This mechanism establishes a heightened threshold for T-cell activation, preventing excessive immune responses at an early stage [43, 46, 47].

The PD-1 pathway represents another critical inhibitory axis, primarily regulating effector T-cell activity in peripheral tissues and within the TME, rather than during the initial priming phase [43, 48, 49]. PD-1 (CD279) is a type I transmembrane glycoprotein belonging to the CD28 superfamily, expressed on activated T cells, B cells, natural killer (NK) cells, monocytes, dendritic cells (DCs), and Tregs [10]. Its primary ligand, PD-L1, also known as CD274 or B7-H1, can be expressed broadly on hematopoietic cells (including Antigen-Presenting Cells (APCs), T cells) and non-hematopoietic cells, including endothelial cells and, significantly, many types of tumour cells [43, 48, 49]. Another ligand, PD-L2 (CD273 or B7-DC), has a more restricted expression pattern, primarily on APCs. When PD-1 on an activated T cell binds to PD-L1 or PD-L2 on another cell (e.g., a tumour cell or APC), it triggers inhibitory signals within the T cell [43, 50]. This signaling entails the recruitment of phosphatases, such as SHP-2, to the cytoplasmic tail of PD-1, which encompasses immunoreceptor tyrosine-based inhibitory motif (ITIM) and immunoreceptor tyrosine-based switch motif (ITSM) domains [43, 51]. The downstream effects include the inhibition of TCR signaling pathways, reduced T-cell proliferation, decreased cytokine production (such as interferon-gamma (IFN-γ)), diminished cytotoxic activity, and potentially the induction of T-cell anergy as a functional unresponsiveness or apoptosis cell death [43, 45, 50]. This mechanism acts to limit immune-mediated damage to peripheral tissues during inflammation and infection, but as discussed later, it is frequently exploited by tumours.

### How cancer exploits immune checkpoint pathways to evade immune detection

Cancer cells employ a complex range of pathways to exploit physiological immune checkpoint pathways, thereby constructing a robust immunosuppressive microenvironment that permits them to evade immune surveillance and destruction. This immune evasion is a hallmark of malignancy and represents a critical barrier to the development of effective anti-tumour immune responses [37, 38, 52, 53].

A principal mechanism by which TME eradication involves the upregulation of immune checkpoint ligands, most notably, PD-L1 on the surface of both cancer cells and non-malignant cells such as TAMs and myeloid-derived suppressor cells (MDSCs), within the TME. The expression of PD-L1 can be driven intrinsically by oncogenic signalling pathways [e.g., via constitutive activation of transcription factors such as STAT3, overexpression of Myelocytomatosis oncogene (MYC), or loss of tumour suppressor Phosphatase and Tensin homolog (PTEN)], or extrinsically in response to inflammatory cytokines, most notably IFN-γ secreted by activated effector T cells within the TME [40, 54].

This upregulation of PD-L1 in response to IFN-γ constitutes an "adaptive resistance" mechanism, forming a negative feedback loop that blunts anti-tumour immunity. Specifically, as tumour-specific cytotoxic T lymphocytes recognize neoplastic cells and secrete IFN-γ, tumour cells respond by increasing PD-L1 expression. PD-L1 then engages PD-1 receptors on activated T cells, transducing inhibitory signals that induce T-cell exhaustion, functional anergy, or apoptosis. This suppresses the cytotoxic activity necessary for effective tumour control [4, 5]. Mechanistically, the engagement of PD-1 inhibits key signaling cascades required for T-cell proliferation, cytokine production, and cell-mediated cytotoxicity, thereby hijacking a pathway that normally protects tissues from chronic inflammatory damage and autoimmunity [40, 55, 56].

In parallel, the CTLA-4 pathway, while predominantly involved in regulating T-cell activation at the level of lymphoid organs, is also commandeered during tumourigenesis. Tumours often foster the recruitment and expansion of Tregs, a subset of CD4+ T cells characterized by the constitutive expression of high levels of CTLA-4 [57, 58]. Within the TME, Tregs exert potent immunosuppressive functions by sequestering and internalizing the co-stimulatory molecules CD80 and CD86 from APCs, depriving effector T cells of critical activation signals [59, 60]. This diversion of the CTLA-4 axis subsequently impairs the priming, proliferation, and effector function of anti-tumour T cells early in the immune response, therefore promoting immune tolerance to tumour antigens and facilitating unchecked tumour progression [57, 61].

Furthermore, Tregs can secrete immunomodulatory cytokines [e.g., Interleukin-10 (IL-10), Transforming growth factor beta (TGF-β)] that further reinforce local immunosuppression. The cumulative effects of these processes include not only the direct inhibition of effector T-cell function through the PD-1/PD-L1 pathway, but also the indirect suppression of anti-tumour immunity via robust Treg-mediated control of initial T-cell activation through the CTLA-4 pathway [61–63].

In summary, malignant cells exploit the PD-1/PD-L1 axis by upregulating PD-L1 to directly inhibit cytotoxic T-cell function within the TME and manipulate the CTLA-4 pathway primarily through the expansion and heightened activity of Tregs, thereby subverting both the effector and initiation phases of the anti-tumour immune response. Collectively, these mechanisms consolidate a profoundly immunosuppressive milieu, enabling tumours to escape immunosurveillance, evade immune-mediated destruction, and continue their neoplastic growth.

## Mechanism of action of immune checkpoint inhibitors (ICIs)

### Blockade of immune checkpoints to restore anti-tumour immunity

ICIs constitute a class of immunotherapeutic agents, predominantly monoclonal antibodies, developed to counteract the immunosuppressive pathways leveraged by malignant cells to evade immune destruction [43, 64]. Their primary mode of action involves targeting inhibitory checkpoint molecules, such as CTLA-4, PD-1, and its ligand, PD-L1. By selectively blocking these molecules, ICIs effectively disengage intrinsic ‘brakes’ on the immune system, permitting reactivation and functional restoration of T lymphocytes capable of recognizing and eradicating tumour cells [44, 64]. Importantly, ICIs act not by directly inducing tumour cell cytotoxicity but by empowering the host’s adaptive immune arsenal, notably T cells, to mediate the anti-tumour response. Rather than exerting direct cytotoxic effects on neoplastic cells, ICIs fundamentally alter the immune landscape, reprogramming the host’s adaptive immune system to become re-engaged in tumour surveillance and destruction [65, 66]. This process predominantly hinges upon the activation and expansion of tumour-specific CD8+ cytotoxic T cells, alongside supportive roles from CD4+ helper T cells. These reinvigorated lymphocytes can mount an adaptive and, often, durable response against malignant cells, occasionally leading to complete or long-lasting tumour regression not commonly seen with conventional cytotoxic chemotherapy [67, 68].

### Inhibition of CTLA-4 pathway

CTLA-4 is a critical immune checkpoint receptor expressed on T cells that modulates the early stages of T-cell activation within secondary lymphoid tissues [69]. It competitively binds to B7 molecules (CD80/CD86) on APCs, thereby antagonizing the co-stimulatory receptor CD28, which is essential for full T-cell activation [70]. Engagement of CTLA-4 recruits the serine/threonine phosphatase PP2A and the tyrosine phosphatase SHP-2 to the cytoplasmic tail of CTLA-4. These phosphatases dephosphorylate key proximal TCR signaling molecules such as cluster of differentiation 3 zeta chain (CD3ζ) and zeta-chain- associated protein kinase 70 (ZAP-70), attenuating downstream activation of the PI3K/AKT/mTOR and RAS/RAF/MEK/ERK (Mitogen-Activated Protein Kinase (MAPK)) cascades [71, 72]. Inhibition of these pathways limits IL-2 production, cell-cycle progression, and metabolic reprogramming required for clonal expansion of effector T cells [72, 73]. The therapeutic monoclonal antibody ipilimumab binds CTLA-4, preventing its interaction with B7 ligands and tipping the balance in favor of CD28-mediated co-stimulatory signaling [64, 74, 75]. As a result, there is a potentiation of T-cell activation, proliferation, and differentiation into effector phenotypes. Other antibodies, such as tremelimumab (an IgG2 monoclonal antibody), also target CTLA-4, interfering with its inhibitory engagement with CD80/CD86 to further augment T-cell responses [76]. This blockade decreases the activation threshold for naive T cells, expanding the repertoire and clonal diversity of tumour-reactive lymphocytes primed by tumour antigen-bearing through APCs [64, 74, 75]. Additionally, anti-CTLA-4 agents contribute to the selective depletion of Tregs within the TME, thereby shifting the immunological balance towards heightened effector T-cell activity [77, 78].

### Blockade of the PD-1/PD-L1 axis

Antibodies targeting the PD-1/PD-L1 immune checkpoint axis (e.g., anti-PD-1 agents nivolumab, pembrolizumab, cemiplimab, dostarlimab, toripalimab; anti-PD-L1 agents atezolizumab, durvalumab, avelumab, envafolimab, sugemalimab) are designed to operate principally within peripheral tissues and the TME [43, 64]. PD-1, an inhibitory receptor expressed primarily on activated T cells, binds its principal ligands PD-L1 and PD-L2, leading to attenuation of T-cell effector functions and promoting T-cell exhaustion under chronic antigenic stimulation [44, 79]. Upon engagement by its ligands PD-L1 or PD-L2, PD-1 cytoplasmic immunoreceptor tyrosine-based inhibitory motif (ITIM) and immunoreceptor tyrosine-based switch-motif (ITSM) become phosphorylated and recruit SHP-2, which directly dephosphorylates CD3ζ, ZAP-70, and the CD28 costimulatory domain. This leads to broad inhibition of the PI3K-AKT survival pathway, suppression of the RAS-RAF-MEK-ERK (MAPK) proliferation pathway, and down-modulation of the Phospholipase C gamma 1 (PLCγ1)- Protein Kinase C (PKC)- Nuclear Factor kappa B (NF-κB) axis [80, 81]. Collectively these events reduce glucose uptake, mitochondrial fitness, cytokine production (IL-2, IFN-γ), and cytotoxic granule release, driving T-cell exhaustion within the TME. PD-1 blockade with antibodies such as nivolumab or pembrolizumab prevents SHP-2 recruitment, reactivating PI3K/AKT and MAPK signaling and restoring effector functions [82].

In addition, checkpoint engagement also interfaces with mammalian target of rapamycin complex ½ (mTORC1/2) complexes, regulating metabolic programs that control glycolysis and fatty-acid oxidation in effector and memory T cells [83]. Negative regulation of Janus Kinase/Signal Transducer and Activator of Transcription (JAK/STAT) signaling dampens transcription of IL-2 and IFN-γ, while downstream suppression of Basic Leucine Zipper ATF-Like transcription factor (BATF), T-box expressed in T cells (T-bet), and cellular myelocytomatosis oncogene (c-Myc) transcription factors contributes to an exhausted phenotype [84–86]. Recent studies also implicate SHP-1, PTEN, and metabolic checkpoints such as AMPK in fine-tuning these responses [87].

Anti-PD-1 antibodies obstruct this interaction by occupying the PD-1 receptor, while anti-PD-L1 antibodies bind PD-L1 expressed on tumour and stromal cells, also preventing engagement with both PD-1 on T cells and, to a lesser extent, CD80 on APCs or tumour cells [43, 79]. This effectively abrogates the inhibitory signal transduction mediated by PD-1, thereby rescuing T cells from dysfunctional or anergic states often induced within the TME after persistent antigen exposure [43, 64]. Restoration of T-cell effector functions increases proliferation, secretion of cytolytic molecules (e.g., perforin, granzymes), and production of pro-inflammatory cytokines such as IFN-γ, ultimately enhancing the capacity for tumour cell lysis [67, 79].

### Activation of T cells and enhancement of the immune response

Blockade of CTLA-4 and PD-1/PD-L1 checkpoints with ICIs orchestrates a multifaceted augmentation of anti-tumour immunity, with core effects cantered on the activation, expansion, and functional reinvigoration of T-cell populations [43]. Inhibiting CTLA-4 during the priming phase within lymphoid tissues increases the pool and diversity of tumour antigen-specific T cells [43], which subsequently traffic to the tumour site. At the tumour location, PD-1/PD-L1 blockade restores the effector capacity of tumour-infiltrating lymphocytes (TILs), counteracting exhaustion induced by chronic antigenic stimulation and sustained PD-L1/PD-1 engagement [43]. Functional restoration encompasses enhanced T-cell proliferation, increased cytotoxicity via upregulation of granzyme and perforin release, and robust IFN-γ production, which further modulates the TME and can promote direct anti-tumour effects [47, 64, 88].

This reinvigoration extends to both CD8⁺ cytotoxic T lymphocytes (CTLs), which directly mediate tumour cell killing, and CD4⁺ helper T cells, which support the broad orchestration of the anti-tumour immune response including sustaining CTL function and activating other immune subsets [47, 64, 79]. Crucially, ICIs preferentially enhance activity within tumour-specific T cells either by promoting initial activation (anti-CTLA-4) or by reversing exhaustion within pre-existing tumour-infiltrating T cells (anti-PD-1/PD-L1). Consequently, the targeted amplification of tumour antigen-directed immunity underlies the durable clinical responses observed in subsets of patients treated with ICIs [43, 64].

### Impact on the tumour microenvironment

The introduction of the ICIs has revolutionized cancer therapy, not only by directly enhancing the cytolytic activity of TILs, but also by fundamentally remodeling the TME to favour antitumour immunity [89, 90]. The TME is a complex and dynamic milieu comprising malignant cells, stromal fibroblasts, endothelial cells, elements of the ECM, and a wide spectrum of immune cells such as CD4⁺ and CD8⁺ T cells, B lymphocytes, NK cells, TAMs, DCs, and MDSCs. Both the quantity and functional state of these populations exert critical control over tumour growth, angiogenesis, immune evasion, and therapy response [91, 92].

#### Quantitative changes in immune cell infiltration and activation

In numerous preclinical and clinical models, ICI therapy especially anti-PD-1 and anti-PD-L1 agents has been shown to increase CD8⁺ T-cell infiltration within tumours. For example, in melanoma patients treated with nivolumab or pembrolizumab, post-treatment biopsies reveal a twofold to fourfold increase in CD8⁺ T-cell density within the TME [93].These reactivated T cells exhibit higher production of IFN-γ, a pleiotropic cytokine that directly inhibits tumour proliferation, augments apoptosis, and upregulates major histocompatibility complex class I MHC class I molecules on tumour cells, enhancing antigen visibility and immunogenicity [43, 47]. IFN-γ further induces the secretion of chemokines such as CXCL9, CXCL10, and CXCL11, which actively recruit additional cytotoxic T cells and NK cells, producing a self-amplifying loop of immune cell infiltration [94]. However, as a counterregulatory measure, IFN-γ is also a key stimulus for induced PD-L1 expression by tumour and stromal cells, contributing to adaptive immune resistance [95].

**Fig. 1:**
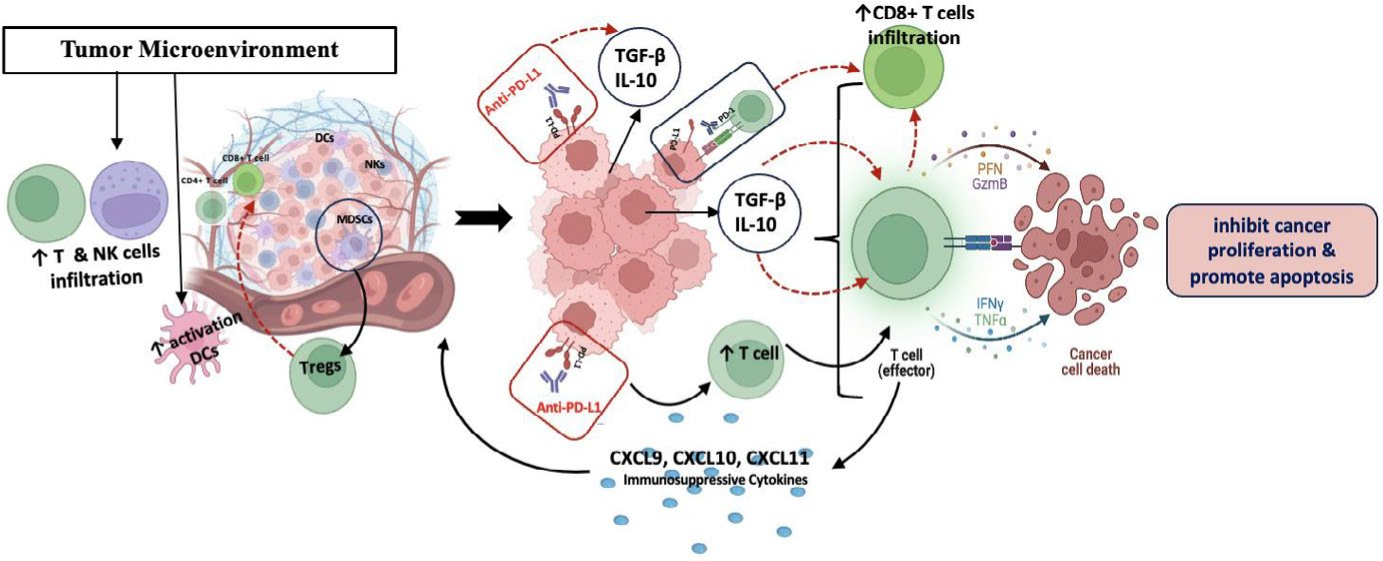
Mechanisms of immune checkpoint inhibitor induced T cell activation and tumour immune modulation within the tumor microenvironment. This figure illustrates the tumor microenvironment's (TME) complex role in anti-tumor immunity. Tumor cells, MDSCs, Tregs, and TAMs within the TME secrete immunosuppressive cytokines like TGF-β and IL-10. These cytokines inhibit effector T cell activity, reducing CD8+ T cell infiltration and hindering cancer cell death, proliferation, and apoptosis, thus promoting cancer progression. The figure also shows immune checkpoint pathways, where PD-L1 on tumor cells inhibits T cells via PD-1. Immune checkpoint blockade (anti-PD-1/PD-L1 therapy) counteracts this, boosting immune cell activity and infiltration, reducing immunosuppressive populations, and ultimately leading to effective anti-tumor immunity and cancer cell apoptosis. However, the persistent presence of immunosuppressive cytokines actively suppresses this beneficial response

The immunological cascade described, including CD8⁺ T-cell infiltration, IFN-γ–mediated antitumour effects, and feedback induction of PD-L1 expression, is schematically illustrated in Fig. 1, providing a visual summary of the dynamic interplay between ICI-induced immune activation and adaptive resistance mechanisms within the TME.

#### Shifts in stromal and myeloid cell phenotypes

ICIs profoundly influence myeloid and stromal cell populations in the TME. Several studies have demonstrated that anti-PD-1 therapy reduces the abundance and suppressive function of MDSCs, which are known to inhibit T-cell function and promote tumour angiogenesis [96]. For example, in non-small cell lung cancer (NSCLC) models, anti-PD-1 treatment leads to a significant (>30%) decrease in intratumoural MDSCs relative to untreated controls [97]. Similarly, ICIs can decrease the frequency and function of Tregs, as evidenced by reduced Forkhead box P3 (FoxP3) + cell densities and diminished expression of suppressive cytokines like IL-10 and TGF-β in responder tissues [98, 99].

Concomitantly, ICIs enhance the maturation of DCs within the TME, as shown by increased expression of co-stimulatory molecules CD80 and CD86; this maturation is crucial for optimal antigen presentation and de novo priming of naïve tumour-specific T cells. TAMs, which in most solid tumours frequently display an immunosuppressive M2-like phenotype, may undergo repolarization towards a proinflammatory M1-like phenotype following ICI therapy, demonstrated by increased expression of inducible nitric oxide synthase (iNOS) and TNF-α, and decreased IL-10 output [100]. This phenotypic plasticity is context-dependent, but studies suggest that in responsive tumours, a higher M1/M2 ratio correlates with improved therapeutic outcomes.

#### Systemic and local immunological consequences

Overall, ICI treatment induces both qualitative and quantitative alterations in the immune landscape within tumour sites. These include increased effector-to-suppressor cell ratios (e.g., CD8⁺ T cells to Tregs), enhanced functional capacity of both cytotoxic and helper T cells, and a shift towards a more inflammatory cytokine and chemokine milieu [101] Importantly, these changes are not restricted to the tumour bed but can also be detected systemically, as evidenced by elevated peripheral effector T cell frequencies and cytokine signatures in patients achieving durable clinical responses.

These multidimensional alterations ultimately render the TME less immunosuppressive and promote effective tumour rejection. Nonetheless, the complexity of cellular crosstalk and the capacity of cancer to evolve adaptive resistance mechanisms (e.g., upregulation of alternative immune checkpoints, metabolic suppression, or stromal fibrosis) underscore the need for ongoing research to further optimize and personalize ICI-based regimens [102, 103].

#### Synergistic effects with other immune cells (e.g., NK cells, macrophages)

Although T cells serve as the main targets and effectors of ICI therapy, a robust anti-tumour immune response depends on the coordinated and synergistic activity of various innate and adaptive immune cell populations [90]. NK cells are innate cytotoxic lymphocytes capable of targeting tumour cells without prior antigen exposure. Similar to T cells, NK cells can express PD-1 and experience functional inhibition via PD-L1 interactions in the TME. Consequently, anti-PD-1/PD-L1 treatments may directly restore NK cell cytotoxicity against cancer cells [104]. Moreover, IFN-γ secreted by reactivated T cells can further stimulate NK cell activity, facilitating cooperative anti-tumour effects between these two lymphocyte lineages [91].

Macrophages, particularly TAMs, are highly prevalent within the TME and can display either tumour-promoting or tumour-suppressive properties depending on their polarization state [93]. ICIs can modulate TAM function; for instance, the cytokine IFN-γ produced by activated T cells can drive macrophages toward an M1-like phenotype, characterized by enhanced phagocytosis, antigen presentation, and release of pro-inflammatory cytokines, all of which support anti-tumour immunity. Furthermore, ICI therapies may suppress the signals that maintain the suppressive M2-like macrophage phenotype [47, 94]. DCs, which are essential antigen-presenting cells for initiating T-cell responses, may also benefit from ICI administration, as this can alleviate PD-L1-mediated inhibition or indirectly stimulate DCs through T cell-derived factors, leading to improved tumour antigen presentation and subsequent T-cell activation [64, 105].

Overall, the multi-faceted interplay between diverse immune cell subsets under ICI therapy highlights the intricate cellular dynamics underlying immune-mediated tumour rejection. Effective immunotherapy often results from mobilizing not only T cells but also engaging additional immune cell types in a cohesive anti-tumour response within the remodeled TME [43, 105]. A detailed understanding of these synergistic mechanisms will inform the development of rational combination strategies to further potentiate the efficacy of ICIs.

#### The immunosuppressive effects of LAG3/TIM-3

Important inhibitory immune checkpoints are Lymphocyte activation gene-3 (LAG-3, CD223) and T-cell immunoglobulin and mucin-domain containing-3 (TIM-3, HAVCR2). LAG-3 and TIM-3 are potent contributors to peripheral tolerance and the regulation of T-cell effector function [106, 107].

CD4 and LAG-3 are structurally related. LAG-3 has a higher affinity than CD4 to bind with major histocompatibility complex class II (MHC-II) resulting in downregulating CD4+ T-cell receptor (TCR) signalling and antigen presentation [108–110]. Over and above binding to MHC-II, other immunosuppressive effects within TME are alternative LAG-3 ligands including galectin-3, fibrinogen-like protein 1 (FGL1), and Liver Sinusoidal Endothelial Cell Lectin (LSECtin) have been implicated in LAG-3 mediated immune suppression [111–113].

Activation of LAG-3 reduces the initiation of TCR-mediated signaling cascades including reduction in cytokine production (e.g., IFN-γ, IL-2), and enhances a transcriptional change associated with T cell dysfunction/exhaustion; a common event within TME, and is co-expressed with PD-1 on chronically stimulated CD8+ T cells, where co-inhibitory signalling causes cooperative clampdown of proliferation and cytotoxicity [113–115].

Overexpression of TIM-3 terminally differentiated effector T cells, regulatory T cells, innate immune cells (including dendritic cells and macrophages), and certain tumour cells, leads to downstream inhibitory effects such as diminished NF-κB and MAPK activation, altered inflammasome activity in myeloid cells, and promotion of T-cell exhaustion [116, 117]. A crucial anticancer immunosuppressive effect of TIM-3 signalling is it contains conserved tyrosine residues within its cytoplasmic tail that, upon ligand binding, deply intracellular adaptor proteins (e.g., HLA-B-associated transcript 3 (Bat3) dissociation, Fyn tyrosine kinase/Tyrosine kinase 2 (Fyn/TYK2) interplay) resulting in downregulation of downstream signalling such as reduced NF-κB and MAPK activation, altered inflammasome activity in myeloid cells, and promotion of T-cell exhaustion [117, 118]. In chronic antigen exposure contexts (viral infection, cancer), sustained TIM-3 expression marks severely exhausted T cells with impaired proliferative capacity and reduced effector cytokine secretion; TIM-3 on myeloid cells can further modulate antigen presentation and produce an immunosuppressive cytokine milieu [119, 120].

## The current clinical applications of ICIs in the fight against cancer

The therapeutic use of ICIs was first clinically realized with the approval of ipilimumab, an antibody directed against CTLA-4, in the treatment of metastatic melanoma [121, 122]. By binding to CTLA-4, ipilimumab interrupts this inhibitory signal, resulting in the amplification of T-cell–mediated immune responses against tumour cells. Ipilimumab has established itself as a cornerstone in the management of advanced melanoma, offering durable survival outcomes and thus altering the previously dismal prognoses for patients with this malignancy. Since its initial approval, ipilimumab has undergone extensive evaluation in numerous oncologic indications beyond advanced melanoma. Over the past decade, clinical trials have demonstrated the utility of ipilimumab, leading to its approval in additional malignancies when used either as monotherapy or in combination with other immune checkpoint inhibitors [123, 124]. A particularly significant advancement involves the combination of ipilimumab with nivolumab, an antibody targeting PD-1. This dual blockade approach harnesses complementary mechanisms: ipilimumab fosters T-cell activation primarily within lymphoid tissues, while nivolumab restores T-cell effector function within the TME. The synergy observed in this combination enhances overall anti-tumour immune responses, translating into improved efficacy across select cancers compared to monotherapies [123, 125]. Notably, the combination of nivolumab and ipilimumab has emerged as a first-line treatment for malignant pleural mesothelioma a rare and aggressive cancer typically associated with asbestos exposure and historically limited therapeutic options. Evidence supporting this approval arose from pivotal phase III clinical trials, such as the CheckMate 743 study, which demonstrated significantly prolonged overall survival in patients receiving the combined immunotherapy regimen versus conventional platinum-based chemotherapy [126, 127].

Subsequent developments of monoclonal antibodies targeting the PD-1/PD-L1 axis. PD-1, thereby reactivating exhausted T cells and restoring their cytotoxic capacity. The clinical benefits of these agents have been substantiated across a broad spectrum of tumour types, including but not limited to melanoma, NSCLC, and refractory Hodgkin lymphoma [127–129].

Importantly, pembrolizumab has received tissue-agnostic approval based on its efficacy in advanced malignancies characterized by specific genomic features, such as microsatellite instability-high (MSI-H), mismatch repair deficiency (dMMR), or a high Tumour Mutational Burden (TMB) [130].This milestone demonstrates the growing emphasis on molecular and genetic profiling in guiding therapeutic decisions, transcending traditional histology-based paradigms. Pembrolizumab has shown pronounced effectiveness in MSI-H/dMMR endometrial and cervical carcinomas, further underscoring its versatility and pivotal role in precision oncology [130, 131].

In addition to PD-1 inhibitors, therapeutic blockade of PD-L1 has emerged as another successful immunotherapeutic strategy. Monoclonal antibodies such as atezolizumab and durvalumab have shown meaningful clinical benefits in several malignancies where PD-L1 expression confers a poor prognosis. These agents have been integrated into treatment algorithms for urothelial carcinoma (bladder cancer), triple-negative breast cancer (TNBC), and unresectable NSCLC. Their ability to disrupt the PD-1/PD-L1 axis has contributed significantly to improved overall survival rates and disease control in patients with otherwise limited options [31, 32, 132–134].

Clinical research has also explored the synergistic potential of dual immune checkpoint blockade. The combination of nivolumab (anti-PD-1) and ipilimumab (anti-CTLA-4) exemplifies this approach and has demonstrated superior efficacy compared to monotherapy in multiple settings, including metastatic melanoma, renal cell carcinoma, and hepatocellular carcinoma. This synergism likely arises from the complementary mechanisms of action, facilitating concurrent activation of various T-cell subpopulations and enhancing antitumour immune responses. Such combination strategies have established new standards of care in several oncologic indications [135–137].

The clinical utility of ICIs continues to grow, as evidenced by the approval of new agents targeting the PD-1 pathway. Cemiplimab has gained prominence as a therapy for cutaneous squamous cell carcinoma, with remarkable objective response rates in this traditionally difficult-to-treat malignancy [138]. Similarly, dostarlimab has demonstrated impressive efficacy in dMMR endometrial cancer, further expanding the therapeutic arsenal against solid tumours [139]. The ongoing expansion of indications and the introduction of novel ICIs underscore their integral role in modern oncology practices.

In aggregate, the evolution of immune checkpoint inhibitors reflects a broader transition toward immunologically driven and genetically informed cancer therapies. These agents have redefined treatment paradigms in multiple tumour types, offering the possibility of durable, and in some cases, curative responses. As research unveils additional immune-modulatory targets and new biomarkers of response, ICIs are poised to remain at the forefront of precision medicine, continuing to revolutionize the management of malignancies through targeted immune modulation.

As summarized in Tables 2 and 3, numerous ongoing and recently completed clinical trials between 2023 and 2025 have expanded the therapeutic landscape of immune checkpoint inhibitors, investigating their efficacy across diverse malignancies, novel combinatorial regimens, and emerging biomarkers of response.

**Table 2:** ICIs clinical trials phase I and II (2023–2025)

NCT ID	ICIs involved	Targeted antigen	Disease	Clinical phase	Status	Estimated enrollment	Study objective	Actual study time/ongoing/completed
NCT05101109	ABL501	LAG-3/PD-L1	Advanced solid tumors	Phase 1/2	Completion 2025	24	To evaluate the safety, tolerability, MTD and/or RP2D, PK, immunogenicity, anti-tumor activity, and the PD effect of ABL501 in subjects with any progressive locally advanced (unresectable) or metastatic solid tumors.	Completed 2025
NCT04986046	Avelumab + Talazoparib	PD-L1/PARP	Breast cancer	Phase 2	Completed 2023	120	To determine the effect of produce prescriptions (vouchers that can be redeemed for produce at participating locations, "FVRx") and a cooking skills program (Home Plate) on dietary quality, food security, perceptions of the food environment, and mental health. Investigators also aim to determine the feasibility and acceptability of the programs.	Completed 2023
NCT05274451	Nivolumab + BMS-986016	PD-1/LAG-3	Gastric cancer	Phase 2	Ongoing	57	To evaluate the safety and tolerability of LYL797, a ROR1-targeted CAR T-cell therapy, in patients with ROR1+ relapsed or refractory triple negative breast cancer (TNBC), non-small cell lung cancer (NSCLC), platinum-resistant epithelial ovarian cancer/ fallopian tube cancer/ primary peritoneal cancer (Ovarian cancer), or Endometrial cancer.	Completion 2024
NCT05108181	Retifanlimab + INCB099280	PD-1/TIGIT	Solid tumors	Phase 1/2	Completed 2024	60	To investigate if the responsiveness to chronic resistance training is dependent on the muscle typology. In a second aim we will investigate the most optimal training frequency for slow-twitchers and fast-twitchers.	Completed 2024
NCT05187000	Cosibelimab + CK-301	PD-1/IL1-2	Cutaneous SCC	Phase 2	Ongoing	30	To define the role of rTMS for the treatment of DOC patients and characterise the neural correlates of its action.	Completion 2025
NCT05291364	Dostarlimab + TSR-033	PD-1/LAG-3	Colorectal cancer	Phase 1/2	Completed	40	To evaluate effect of addition of dexmedetomidine as an adjuvant to alcohol and local anesthetics for chemical neurolysis to control pain in patients with intra-abdominal malignancy.	Completion 2025
NCT06413017	PD-1 or PD-L1 inhibitors	PD-1, PD-L1	Hepatocellular Carcinoma (HCC)	Phase 2	Recruiting	30	Evaluate efficacy of nimotuzumab combined with PD-1 or PD-L1 inhibitors in advanced HCC after first-line treatment failure.	Ongoing
NCT06020651	ICIs	N/A	Renal Cell Carcinoma, Bladder Cancer, MSI-H Cancer, Cancer	N/A	Recruiting	40	The VICKI study aims at furthering our knowledge on the mechanisms of atherosclerotic plaque instability by means of a prospective single-centre pilot study, by comparing: 1- surrogate markers of clinical vasculo-toxicity with arterial Doppler (flow mediated reserve) as defined by the International Cardio-Oncology Society. 2- circulating biomarkers. Before and after receiving ICIs for solid cancer treatment.	Ongoing
NCT06923020	ICIs	N/A	Gastric Neoplasm, Esophageal Adenosquamous Carcinoma	N/A	Enrolling by invitation	200	To explore the clinical significance of nutrition-related prognostic indicators in immunotherapy by evaluating nutritional status and comparing treatment efficacy of first-line immune checkpoint inhibitors among advanced gastric cancer and esophageal cancer patients (including unresectable locally advanced, recurrent, or metastatic gastrointestinal cancers such as gastric/gastroesophageal junction adenocarcinoma and esophageal carcinoma) with different nutritional profiles.	
NCT06814548	Anti-PD-1/PD-L1 Agents first line setting, & ICI-based second-line therapy.	PD-1/PD-L1	Gastric Cancer, Gastroesophageal Junction Cancer.	N/A	Not Yet Recruiting	200	To evaluate the efficacy and safety of retreatment with immune checkpoint inhibitors (ICIs) in patients with advanced gastric cancer (GC) or gastroesophageal junction cancer (GEJC) who previously received ICIs but progressed.	Ongoing
NCT04975932	Atezolizumab, pembrolizumab, nivolumab, camrelizumab, tislelizumab, sintilimab or other ICIs	PD-1/PD-L1	Hepatocellular Carcinoma	N/A	Completed	826	To evaluate the safety and efficacy of transarterial chemoembolization (TACE) in combination with immune checkpoint inhibitors (ICIs) in patients with hepatocellular carcinoma (HCC).	Completed
NCT06119347	Pembrolizumab, Sintilimab, toripalimab, Camrelizumab, Tislelizumab.	PD-1	AKI Incidence of Cancer Patients Receiving AntiVEGF or ICIs.	N/A	Completed	1581	To compare the AKI events in cancer patients receiving anti-vascular endothelial growth factor monoclonal antibody (AntiVEGF) vs immune checkpoint inhibitors (ICIs). The main question it aims to answer is whether the choice between AntiVEGF and ICIs affects the risks of acute kidney injury in cancer patients.	Completed
NCT06632106	PD-1 inhibitors, Lenvatinib, Camrelizumab, Sintilimab, Tislelizumab, Pembrolizumab, Atezolizumab	PD-1/PD-L1	Hepatic Arterial Infusion, hepatocellular Carcinoma (HCC)	N/A	Active Not Recruiting	97	To evaluate the safety and efficacy of hepatic arterial infusion chemotherapy (HAIC) in combination with PD-1 inhibitors and Lenvatinib in patients with different tumor burden advanced-stage hepatocellular carcinoma (HCC) with portal vein tumor thrombus (PVTT).	Ongoing
NCT04352335	ICIs	N/A	Advanced Lung Cancer	N/A	Completed	53	To evaluate the change of neutrophil to lymphocyte ratio (NLR) after 6-week treatment of immune checkpoint inhibitors (ICIs) with or without immunomodulatory drugs and recognize the effect of post-treatment NLR and overall survival in advanced lung cancer patients by retrospective review.	Completed
NCT05108623	Nivolumab, pembrolizumab	PD-1	Tumor, Solid	Phase 1	Completed	34	Explore the safety, tolerability, and preliminary clinical activity of agenT-797 in combination with approved immune checkpoint inhibitors (ICIs), including pembrolizumab and nivolumab, in participants with r/r solid tumors.	Completed
NCT05934214	Nivolumab, pembrolizumab, cemiplimab, dostarlimab, durvalumab, atezolizumab, avelumab, ipilimumab, tremelimumab, relatlimab	PD-1/PD-L1, CTLA-4, LAG-3	Cancer|Immune-related Adverse Event|Immune Checkpoint Inhibitor-Related Myocarditis	N/A	Completed	141630	To characterize immune-related adverse reactions associated with immune-checkpoint inhibitors, particularly their time-to-onset, co-occurrence, factors associate with their over-report and fatality.	Completed
NCT05688046	PD-1/ PDL-1 inhibitors	PD-1/ PDL-1	Non-small cell lung cancer (NSCLC)	N/A	Completed	79	To evaluate the efficacy and safety of ICI + angiogenesis inhibitor combinations in elderly NSCLC patients.	Completed
NCT05942872	Ipilimumab, pembrolizumab, nivolumab, cemiplimab	PD-1, CTLA-4	Paraneoplastic Syndromes	N/A	Completed	90	To characterize the main clinical and paraclinical features of NirAEs in a large cohort of NirAE patients, to assess long-term outcomes and to identify prognostic factors. This study will help define new guidelines regarding NirAE prediction and management.	Completed
NCT05142709	PD-1/ PDL-1 inhibitors	PD-1/PD-L1	Metastatic Esophageal Squamous Cell Carcinoma	N/A	Active Not Recruiting	600	To evaluate the real-world effectiveness and safety of combining ICIs (PD-1 inhibitors) with chemotherapy in patients with advanced or metastatic ESCC, outside the controlled setting of clinical trials.	Ongoing
NCT02724488	ICIs	N/A	Head and Neck Cancer, Melanoma	14	Active Not Recruiting	14	1-To investigate the feasibility of performing ultra-deep sequencing of plasma derived circulating tumor DNA (ctDNA) in individual patients with advanced solid tumors who are currently being treated with immune checkpoint inhibitors (ICIs). 2- To investigate the clonal evolution of tumors under the selection pressure of ICIs.	Ongoing
NCT04566432	ICIs	N/A	Lung Neoplasms|Lung Cancer, Nonsmall Cell|Adenocarcinoma of Lung|Squamous Cell Lung Cancer	N/A	Recruiting	250	To evaluate the predictive value of ctDNA in response, relapse for patients treated with immune checkpoint inhibitors or targeted therapy for ALK, ROS1, MET ex14 skipping.	Ongoing
NCT05219851	ICIs	N/A	Cancer Patients	N/A	Recruiting	250	To collect data on the risk factors for acute radiation pneumonitis in patients with prior receipt of immune checkpoint inhibitors.	Ongoing
NCT05821751	Nivolumab, pembrolizumab	PD-1	Head and Neck Squamous Cell Carcinoma	N/A	Recruiting	40	To characterize the translational and clinical implications of the regular assumptions of inulin on Gut Microbiota, circulating cytokines and immune cells dynamics during ICIs +/- chemotherapy on patients affected by R/M HNSCC.	Ongoing
NCT06461338	ICIs	N/A	Non-small Cell Lung Cancer|Non-Small-Cell Lung Carcinoma	N/A	Not Yet Recruiting	60	Evaluates the effect and safety of integrating acupuncture with immunotherapeutic sensitization in treating NSCLC.	Ongoing
NCT06975384	ICIs	N/A	Lung Cancer	N/A	Recruiting	1270	To explore the associations of sleep disturbance with progression, efficacy of immune checkpoint inhibitors (ICIs) and prognosis of Lung Cancer.	Ongoing
NCT05477979	ICIs	N/A	Lung Cancer, Psychological Stress	N/A	Recruiting	750	To explore the associations of psychological stress with progression, efficacy of immune checkpoint inhibitors (ICIs) and prognosis of Lung Cancer.	Ongoing
NCT06446154	Sintilimab	N/A	Hepatocellular Carcinoma|Immune Checkpoint Inhibitors|Anti-angiogenic Therapy|Second-line Treatment	Phase 2	Recruiting	36	To explore the efficacy and safety of fruquintinib as second-line treatment for patients with unresectable HCC previously treated with immune checkpoint inhibitors.	Ongoing
NCT06904573	Pembrolizumab, toripalimab.	N/A	Advanced Urothelial Carcinoma	Phase 2	Recruiting	222	Evaluating the Efficacy and Safety of Immunotherapy Combined With Oral Probiotics Compound (Biolosion) in Patients With Advanced Urothelial Carcinoma.	Ongoing
NCT06163820	Ipilimumab, nivolumab	N/A	Melanoma Brain Metastases	Phase 1 / 2	Recruiting	46	To evaluate the safety and efficacy of the combination of bevacizumab, with ipilimumab plus nivolumab, and hypofractionated stereotactic radiotherapy (hSRT) in patients with symptomatic melanoma brain metastases (MBM).	Ongoing
NCT06508034	Ipilimumab, Anti-PD-1/PD-L1 inhibitors	CTLA-4, PD-1/PD-L1	Malignant Solid Neoplasm	N/A	Recruiting	30	To evaluate the incidence of immune checkpoint inhibitors (ICI)-induced colitis (IIC) in patients with solid malignancies receiving over-the-counter multi-strain probiotic blend and ICIs in both cohorts: (1) anti-Cytotoxic T lymphocyte-associated protein-4 (CTLA-4) +/- anti-programmed cell death-1 (PD-1)/programmed cell death-1 ligand 1 (PD-L1), and (2) anti-PD-1/PD-L1 +/- chemo.	Ongoing
NCT06629714	First-line ICIs	N/A	Non-Small Cell Lung Cancer, Gastroesophageal Cancer.	N/A	Active Not Recruiting	180	To explore the impact of pretreatment emotional distress on survival and the predictive role of peripheral blood metabolic and inflammatory markers in immunotherapy response among treatment-naïve, advanced and inoperable Gastroesophageal Cancer (GEC) and non-small-cell lung cancer (NSCLC).	Ongoing
NCT03732664	Nivolumab, Pembrolizumab, Toripalimab, Sintilimab, Sintilimab, Tislelizumab, Durvalumab	PD-1/PD-L1	Resectable NSCLC	Phase 1	Completed	27	Evaluate the safety and feasibility of preoperative administration nivolumab in patients with high-risk resectable NSCLC, and will facilitate a comprehensive exploratory characterization of the tumor immune milieu and circulating immune cells and soluble factors in these patients.	Completed
NCT06435013	PD-1/PD-L1 inhibitors	PD-1/PD-L1	Hepatocellular Carcinoma	N/A	Completed	208	To compare the efficacy and safety of two treatment approaches in unresectable HCC: Lenvatinib (TKI) + Immune Checkpoint Inhibitors (ICIs) & Bevacizumab (anti-VEGF) + ICIs + Hepatic Arterial Infusion Chemotherapy (HAIC).	Completed
NCT06669377	N/A	N/A	Hepatocellular Carcinoma	N/A	Recruiting	444	To assess the safety and efficacy of transcatheter arterial chemoembolization (TACE) combined with immune checkpoint inhibitors (ICIs) plus molecular targeted therapy (MTT) after irradiation stent placement (ISP) as first line treatment for HCC patients with Vp4 PVTT.	Ongoing
NCT06277674	Cadonilimab	CTLA-4, PD-1/PD-L1	Non-small Cell Lung Cancer (NSCLC)	Phase 2	Recruiting	20	This study was designed to evaluate the efficacy and safety of cadonilimab (anti PD-1 and CTLA-4 bispecific antibody) in combination with pemetrexed and anlotinib for treatment of elderly patients with T790M-negative advanced non-squamous non-small cell lung cancer following resistance to EGFR-TKI.	Ongoing
NCT06926179	PD-1/PD-L1 inhibitors	PD-1/PD-L1	Non-small Cell Lung Cancer (NSCLC)	N/A	Recruiting	500	To evaluate the safety, efficacy and survival outcomes of neoadjuvant/induction immunotherapy in patients with non-small cell lung cancer (NSCLC).	Ongoing
NCT06066216	Cadonilimab	CTLA-4, PD-1/PD-L1	Endometrial Cancer	Phase 2	Not Yet Recruiting	45	To evaluate objective response rate of cadonilimab plus chemotherapy.	Ongoing
NCT06238752	Tislelizumab	PD-1	Advanced Gastric Adenocarcinoma	Phase 2	Completed	33	Explore the efficacy and safety of a combination therapy regimen with antiangiogenic agent (apatinib), ICI (tislelizumab), and chemotherapy (capecitabine+ Oxaliplatin, XELOX) as first-line treatment for HER2-negative, advanced G/GEJ cancer patients with signet ring cell carcinoma or peritoneal metastasis.	Completed
NCT05812274	PD-1/PD-L1 inhibitors	PD-1/PD-L1	Non-Small Cell Lung Cancer (NSCLC)	N/A	Active Not Recruiting	280	To study the effectiveness of the AprictyRxTM care service to improve treatment outcomes of ethnic/racial minority N.S.C.L.C. patients receiving standard of care immunotherapy, and reduce the frequency of healthcare system interactions.	Ongoing
NCT05599789	Pembrolizumab	PD-1	Non-Small Cell Lung Cancer Metastatic	Phase 2	Active Not Recruiting	47	To evaluate the efficacy of the triplet combination (pembrolizumab + plinabulin + docetaxel) in patients with metastatic non-small cell lung cancer (NSCLC) who progressed after prior ICI therapy.	Ongoing
NCT05713994	Sintilimab, camrelizumab|	PD-1	Hepatocellular Carcinoma Non-resectable	N/A	Recruiting	300	To evaluate the efficacy, prognosis, adverse effects, and factors for predicting therapeutic effects and clinical prognosis of combined therapy of hepatic artery infusion chemotherapy (HAIC), tyrosine kinase inhibitor/ anti-VEGF antibody, and anti-PD-1/ PD-L1 antibody for advanced hepatocellular carcinoma which initially unsuitable for the radical therapy, including resection, transplantation, or ablation.	Ongoing
NCT05717738	Anti-PD-1 monoclonal antibody, sintilimab Atezolizumab, camrelizumab	PD-1/PD-L1	Hepatocellular Carcinoma Non-resectable	N/A	Recruiting	300	The aim of this study is to the efficacy, prognosis, adverse effects, and factors for predicting therapeutic effects and clinical prognosis of combined therapy of transarterial chemoembolization (TACE), Anti-VEGF antibodies or pan-target anti-angiogenic drugs, and anti-PD-1/ PD-L1 antibody for advanced hepatocellular carcinoma which initially unsuitable for the radical therapy, including resection, transplantation, or ablation.	Ongoing
NCT03645928	Pembrolizumab, Ipilimumab, Nivolumab, relatlimab.	PD-1, CTLA-4	Metastatic Melanoma, Squamous Cell Carcinoma of the Head and Neck, Non-small Cell Lung Cancer	Phase 2	Recruiting	245	To evaluate the safety and efficacy of LN-145, an adoptive cell therapy using autologous tumor-infiltrating lymphocytes (TILs), in patients with advanced solid tumors.	Ongoing
NCT06321640	Atezolizumab	N/A	Breast Cancer, Lung Cancer, Melanoma, Head and Neck Cancer, Urothelial Carcinoma, Colorectal Cancer, Liver Metastases, Lung Metastases	Phase 1 / 2	Recruiting	265	To assess safety, tolerability, preliminary efficacy, and pharmacokinetics (PK) of cabozantinib taken in combination with atezolizumab in subjects with multiple tumor types, including advanced urothelial carcinoma (UC) (including bladder, renal pelvis, ureter, urethra), renal cell carcinoma (RCC), castration-resistant prostate cancer (CRPC), non-small-cell lung cancer (NSCLC), triple negative breast cancer (TNBC), ovarian cancer (OC), endometrial cancer (EC), hepatocellular cancer (HCC), gastric cancer/gastroesophageal junction cancer/lower esophageal cancer (GC/GEJC/LEC), colorectal cancer (CRC), head and neck (H&N) cancer, and differentiated thyroid cancer (DTC).	Ongoing

**Table 3:** ICIs clinical trials phase III (2023 -2025)

NCT ID	ICIs involved	Targeted antigen	Disease	Clinical phase	Status	Estimated enrollment	Study objective	Actual study time/ongoing/completed
NCT05194709	Pembrolizumab + Lenvatinib	PD-1/VEGFR	Hepatocellular carcinoma	Phase 3	Completed 2023	40	to evaluate the safety, tolerability, initial efficacy and pharmacokinetics (PK) of anti-5T4 CAR-NK cells in patients with advanced solid tumors.	Completed 2023
NCT04736173	Nivolumab + Relatlimab	PD-1/LAG-3	Advanced melanoma	Phase 3	Ongoing	169	investigate the efficacy and safety of combination therapy with dom + zim compared with pembrolizumab monotherapy in patients with PD-L1-high NSCLC.	Completion 2024
NCT05015712	Dostarlimab + Niraparib	PD-1/PARP	Endometrial cancer	Phase 3	Ongoing	66	A randomized controlled study investigating the efficacy of moderate-intensity continuous training (MICT) on improving cardiopulmonary function in patients after transcatheter aortic valve replacement (TAVR).	Completion 2025
NCT04988802	Atezolizumab + Tiragolumab	PD-L1/TIGIT	NSCLC	Phase 3	Completed 2023	37	To investigate this potential interaction between dietary supplements used in pregnancy and levothyroxine absorption in order to test the safety of the use of these preparations in pregnant women who are on replacement therapy with levothyroxine.	Completed 2023
NCT05239728	Durvalumab + Tremelimumab	PD-L1/CTLA-4	Biliary tract cancer	Phase 3	Ongoing	1800	To assess the efficacy and safety of oral belzutifan (MK-6482) plus intravenous (IV) pembrolizumab (MK-3475) compared to placebo plus pembrolizumab, in the adjuvant treatment of Clear Cell Renal Cell Carcinoma (ccRCC) post nephrectomy.	Completion 2025
NCT04961307	Ipilimumab + Nivolumab	CTLA-4/PD-1	Mesothelioma	Phase 3	Completed 2023	30	To evaluate the effect of trastuzumab on cardiac function; determine the sensitivity of echocardiography and biomarker indicators And specificity, explore effective and specific early warning indicators, and provide technical support for the evaluation of the cardiac safety of anti-tumor drugs.	Completed 2023
NCT04256421	Tiragolumab (TIGIT) + Atezolizumab (PD-L1)	TIGIT, PD-L1	Small Cell Lung Cancer (SCLC)	Phase 3	Active, not recruiting	490 (Actual)	Assess efficacy and safety of Tiragolumab combined with Atezolizumab in SCLC.	Ongoing
NCT05064059	Favezelimab (LAG-3) + Pembrolizumab (PD-1)	LAG-3, PD-1	PD-1 Positive Colorectal Cancer	Phase 3	Active, not recruiting	441/505	Compare coformulated Favezelimab/Pembrolizumab (MK-4280A) with standard care (Regorafenib/TAS-102) in metastatic PD-L1 positive colorectal cancer.	Completed
NCT03358875	BGB-A317 (PD-1)	PD-1	Non-Small Cell Lung Cancer (NSCLC)	Phase 3	Active, not recruiting	805	Evaluate efficacy and safety of BGB-A317 in NSCLC.	Completed
NCT04294810	Tiragolumab (TIGIT) + Atezolizumab (PD-L1)	TIGIT, PD-L1	Non-Small Cell Lung Cancer (NSCLC)	Phase 3	Active, not recruiting	620/635	Assess efficacy and safety of Tiragolumab combined with Atezolizumab in NSCLC.	Ongoing
NCT04746924	Tislelizumab (PD-1) + Ociperlimab (TIGIT)	PD-1, TIGIT	Non-Small Cell Lung Cancer (NSCLC)	Phase 3	Active, not recruiting	662	Compare Tislelizumab and Ociperlimab combination with Pembrolizumab in untreated PD-L1 high NSCLC.	Ongoing
NCT05568095	Domvanalimab (TIGIT) + Zimberelimab (PD-1)	TIGIT, PD-1	Upper Gastrointestinal Tract Adenocarcinoma (UGTA)	Phase 3	Active, not recruiting	1040/970	Evaluate Domvanalimab and Zimberelimab combination with chemotherapy versus Nivolumab and chemotherapy in UGTA.	Ongoing
NCT04266301	MBG453 (TIM-3) + Azacitidine	TIM-3	Myelodysplastic Syndromes (MDS) / Chronic Myelomonocytic Leukemia (CMML-2)	Phase 3	Active, not recruiting	530 (Actual)	Assess efficacy and safety of MBG453 in combination with Azacitidine in MDS/CMML-2.	Completed
NCT06726421	ICIs	N/A	Renal Cell Carcinoma, Kidney Cancer Metastatic, Renal Cell Carcinoma Metastatic	Phase3	Recruiting	252	To compare the progression-free survival (PFS) between patients receiving SBRT + standard systemic therapy versus standard systemic therapy alone.	Ongoing
NCT04128696	Feladilimab (ICOS) + Pembrolizumab (PD-1)	ICOS, PD-1	Head and Neck Squamous Cell Carcinoma (HNSCC)	Phase 2/3	Active, not recruiting	315/600	Evaluate if adding Feladilimab to Pembrolizumab improves efficacy in PD-L1 positive recurrent/metastatic HNSCC.	
NCT05123482	Pembrolizumab + Favezelimab	PD-1/LAG-3	Renal cell carcinoma	Phase 2/3	Ongoing	370	Studying a new compound, AZD8205, as a possible treatment for advanced or metastatic solid tumours alone or in combination with anti-cancer agents.	
NCT05194709	Pembrolizumab + Lenvatinib	PD-1/VEGFR	Hepatocellular carcinoma	Phase 3	Completed 2023	40	to evaluate the safety, tolerability, initial efficacy and pharmacokinetics (PK) of anti-5T4 CAR-NK cells in patients with advanced solid tumors.	Completed 2023
NCT04736173	Nivolumab + Relatlimab	PD-1/LAG-3	Advanced melanoma	Phase 3	Ongoing	169	Investigate the efficacy and safety of combination therapy with dom + zim compared with pembrolizumab monotherapy in patients with PD-L1-high NSCLC.	Completion 2024
NCT05015712	Dostarlimab + Niraparib	PD-1/PARP	Endometrial cancer	Phase 3	Ongoing	66	A randomized controlled study investigating the efficacy of moderate-intensity continuous training (MICT) on improving cardiopulmonary function in patients after transcatheter aortic valve replacement (TAVR).	Completion 2025
NCT04988802	Atezolizumab + Tiragolumab	PD-L1/TIGIT	NSCLC	Phase 3	Completed 2023	37	To investigate this potential interaction between dietary supplements used in pregnancy and levothyroxine absorption in order to test the safety of the use of these preparations in pregnant women who are on replacement therapy with levothyroxine.	Completed 2023
NCT05239728	Durvalumab + Tremelimumab	PD-L1/CTLA-4	Biliary tract cancer	Phase 3	Ongoing	1800	To assess the efficacy and safety of oral belzutifan (MK-6482) plus intravenous (IV) pembrolizumab (MK-3475) compared to placebo plus pembrolizumab, in the adjuvant treatment of Clear Cell Renal Cell Carcinoma (ccRCC) post nephrectomy.	Completion 2025
NCT04961307	Ipilimumab + Nivolumab	CTLA-4/PD-1	Mesothelioma	Phase 3	Completed 2023	30	To evaluate the effect of trastuzumab on cardiac function; determine the sensitivity of echocardiography and biomarker indicators And specificity, explore effective and specific early warning indicators, and provide technical support for the evaluation of the cardiac safety of anti-tumor drugs.	Completed 2023
NCT04256421	Tiragolumab (TIGIT) + Atezolizumab (PD-L1)	TIGIT, PD-L1	Small Cell Lung Cancer (SCLC)	Phase 3	Active, not recruiting	490 (Actual)	Assess efficacy and safety of Tiragolumab combined with Atezolizumab in SCLC.	Ongoing
NCT05064059	Favezelimab (LAG-3) + Pembrolizumab (PD-1)	LAG-3, PD-1	PD-1 Positive Colorectal Cancer	Phase 3	Active, not recruiting	441/505	Compare coformulated Favezelimab/Pembrolizumab (MK-4280A) with standard care (Regorafenib/TAS-102) in metastatic PD-L1 positive colorectal cancer.	Completed
NCT03358875	BGB-A317 (PD-1)	PD-1	Non-Small Cell Lung Cancer (NSCLC)	Phase 3	Active, not recruiting	805	Evaluate efficacy and safety of BGB-A317 in NSCLC.	Completed
NCT04294810	Tiragolumab (TIGIT) + Atezolizumab (PD-L1)	TIGIT, PD-L1	Non-Small Cell Lung Cancer (NSCLC)	Phase 3	Active, not recruiting	620/635	Assess efficacy and safety of Tiragolumab combined with Atezolizumab in NSCLC.	Ongoing
NCT04746924	Tislelizumab (PD-1) + Ociperlimab (TIGIT)	PD-1, TIGIT	Non-Small Cell Lung Cancer (NSCLC)	Phase 3	Active, not recruiting	662	Compare Tislelizumab and Ociperlimab combination with Pembrolizumab in untreated PD-L1 high NSCLC.	Ongoing
NCT05568095	Domvanalimab (TIGIT) + Zimberelimab (PD-1)	TIGIT, PD-1	Upper Gastrointestinal Tract Adenocarcinoma (UGTA)	Phase 3	Active, not recruiting	1040/970	Evaluate Domvanalimab and Zimberelimab combination with chemotherapy versus Nivolumab and chemotherapy in UGTA.	Ongoing
NCT04266301	MBG453 (TIM-3) + Azacitidine	TIM-3	Myelodysplastic Syndromes (MDS) / Chronic Myelomonocytic Leukemia (CMML-2)	Phase 3	Active, not recruiting	530 (Actual)	Assess efficacy and safety of MBG453 in combination with Azacitidine in MDS/CMML-2.	Completed
NCT06726421	ICIs	N/A	Renal Cell Carcinoma, Kidney Cancer Metastatic, Renal Cell Carcinoma Metastatic	Phase3	Recruiting	252	To compare the progression-free survival (PFS) between patients receiving SBRT + standard systemic therapy versus standard systemic therapy alone.	Ongoing

## Immune-related adverse events following checkpoint inhibition: mechanistic basis and management strategies

As previously discussed, ICIs targeting PD-1, PD-L1, and CTLA-4 have revolutionized cancer therapy but can disrupt immune self-tolerance, resulting in immune-related adverse events (irAEs). These toxicities are now recognized as on-target consequences of sustained immune activation, reflecting the same immunostimulatory mechanisms that drive durable antitumour responses [140, 141]. Understanding how these toxicities arise at the molecular and cellular levels is therefore critical for balancing efficacy with safety.

At the mechanistic level, ICI therapy can trigger irAEs through several mechanisms. T-cell–mediated responses arise from cross-reactivity between tumour and self-antigens or expansion of pre-existing autoreactive T cells, leading to tissue inflammation such as uveitis [141, 142]. Bystander inflammation involves nonspecific cytokine release and immune cell infiltration causing collateral injury (e.g., retinal vasculitis) [140, 143, 144]. Autoantibody-mediated mechanisms reflect ICI-driven B-cell activation and autoantibody production, potentially exacerbating paraneoplastic syndromes [145, 146]. Together, these pathways illustrate systemic immune disinhibition underlying ICI toxicity. Recent multi-omic analyses have revealed shared transcriptional and cytokine profiles between irAEs and classical autoimmune diseases, implicating IFN-driven gene signatures, HLA polymorphisms, and gut microbiota composition as modulators of susceptibility [147, 148].

Clinically, irAEs can affect nearly any organ, with the skin, gastrointestinal tract, endocrine glands, liver, and lungs most frequently involved. Cutaneous and endocrine toxicities are often indolent and manageable, whereas myocarditis, pneumonitis, and neurologic syndromes, though rare, are potentially fatal [149]. Notably, irAEs may correlate with improved tumour control in some settings, suggesting a shared immunogenic trajectory between antitumour and autoimmune responses [150, 151].

These clinical patterns underscore the need for vigilant monitoring and timely therapeutic intervention, as the balance between effective antitumour immunity and immune-mediated toxicity is often delicate. Consequently, a structured, multidisciplinary approach to irAE management integrating early detection, precise grading, and mechanistic insight has become essential to mitigate morbidity while preserving oncologic benefit. Management requires early recognition, precise grading, and coordinated intervention. Mild (grade 1) events may resolve with supportive care and continued ICI therapy, while moderate to severe (grade ≥ 2) toxicities necessitate temporary discontinuation and corticosteroids (prednisone 0.5–2 mg/kg/day or IV methylprednisolone for grade 3–4) [152, 153]. Steroid-refractory cases are treated with second-line immunomodulators such as infliximab (anti-TNF) for colitis, mycophenolate mofetil for hepatitis, IVIG or rituximab for neurologic or hematologic events, and tocilizumab (anti-IL-6R) for cytokine-driven syndromes. Multidisciplinary surveillance and biomarker-guided risk stratification encompassing baseline autoantibody screening, cytokine and soluble immune factor profiling, and gut microbiome characterization are increasingly recognized as key components of proactive irAE prediction and management [154–157].

## Current insights into mechanisms of cancer cell resistance to immunotherapy

Despite the remarkable clinical success of ICIs, only a fraction of patients achieves durable responses, while many display primary resistance (lack of initial benefit) or develop acquired resistance (relapse after an initial response). Understanding these mechanisms is essential for biomarker discovery and the rational design of next-generation therapies.

With the increasing application of ICIs in clinical practice, investigators have sought robust biomarkers to identify patients most likely to respond. The rationale for predictive biomarker development stems from the observation that only a subset of patients achieves meaningful and sustained responses, while others demonstrate primary or acquired resistance [158]. Early and ongoing efforts have focused on the analysis of PD-L1 expression on tumour and immune cells using immunohistochemical assays. The level of PD-L1, measured as tumour proportion score (TPS) or as a combined positive score (CPS) which takes into account both tumour and infiltrating immune cells has been correlated with response rates in several tumour types, most notably NSCLC and head and neck squamous cell carcinoma (HNSCC) [159, 160]. Nevertheless, a substantial proportion of patients either fail to respond to immune checkpoint inhibitors or experience disease progression following an initial response, and this phenomenon of resistance has become a major focus of intensive scientific research aimed at elucidating its underlying mechanisms and developing strategies to overcome it.

### Primary (intrinsic) resistance

Primary (intrinsic) resistance occurs before treatment and prevents any meaningful initial response.

#### Tumour antigenicity and antigen presentation

One of the central features of primary resistance lies in the intrinsic immunogenicity of the tumour. Tumours characterized by a low TMB or lacking immunogenic neoantigens are often less capable of eliciting cytotoxic immune responses [161]. Without sufficient neoantigen presentation, even highly active T cells are unable to adequately recognize and target malignant cells, leading to immunotherapy failure from the outset [161, 162]. Moreover, defects in the antigen processing and presentation machinery, especially mutations in genes involved in the major histocompatibility complex (MHC) class I pathway or beta-2-microglobulin (B2M), further compromise immune recognition [162].

#### Immune-excluded (“cold”) tumour microenvironment (TME)

**Fig. 2:**
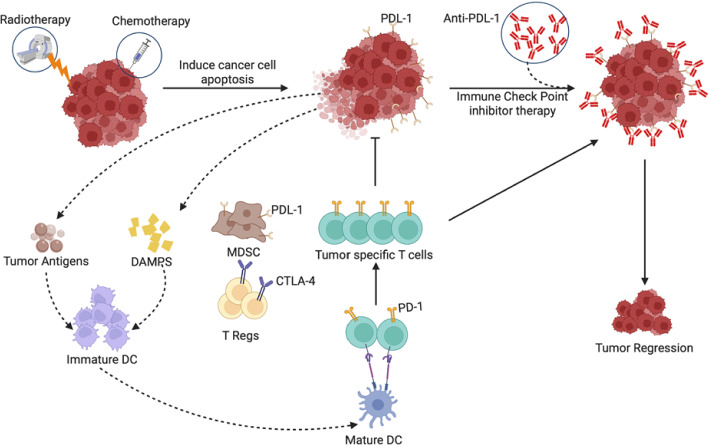
Mechanisms of immune response in immune checkpoint inhibitor (ICI) therapy and the rationale for combination strategies. Traditional cancer treatments, such as chemotherapy and radiotherapy, not only induce apoptosis in cancer cells but also release tumor-associated antigens and various damage-associated molecular patterns (DAMPs) that activate dendritic cells (DCs). The interactions between cancer cells and activated DCs facilitate the cross-priming and recruitment of tumor-specific T cells, leading to enhanced T cell-mediated destruction of tumor cells. However, conventional cancer therapies may also induce immunosuppression within the TME resulting in resistance to immunotherapy through mechanisms such as the upregulation of PD-L1 on tumor cells and the accumulation of myeloid-derived suppressor cells (MDSCs) and regulatory T cells (Tregs). Immune checkpoint inhibitors (ICIs) can rejuvenate the function of exhausted T cells or amplify the activity of cytotoxic CD8+ T cells, thereby restoring antitumor immune responses

TME factors also play a fundamental role in establishing primary resistance. ‘Cold’ or non-inflamed tumours often exhibit a paucity of TILs and overexpress immunosuppressive cytokines such as TGF-β and IL-10, which hinder effective T cell infiltration [163, 164] (Fig. 2). Additionally, the enrichment of immunosuppressive cell types, particularly Tregs, MDSCs, and TAMs, directly suppresses effector cell function and facilitates immune evasion [165, 166].

#### Oncogenic pathway activation

Genetic and epigenetic alterations can also foster immune resistance. Oncogenic signaling pathways, such as activation of WNT/β-catenin, PTEN loss, or MAPK pathway mutations have been shown to result in T cell exclusion from tumours or suppress interferon signaling, further insulating cancer cells from immune attack [167, 168]. These tumour-intrinsic mechanisms can create an immune-excluded phenotype regardless of systemic immune competency.

### Acquired (secondary) resistance

Beyond primary resistance, many tumours experience disease progression after an initial response to ICIs a phenomenon known as acquired (secondary) resistance. Tumours may evade immune pressure through the outgrowth of antigen-loss variants, whereby immune-recognized epitopes are downregulated or lost from the tumour cell surface [168]. Parallel to this, secondary mutations in genes involved in IFN-γ signaling pathways, such as JAK1/2, can render tumours resistant to immune effector mechanisms [167].

Acquired resistance also encompasses the upregulation of non-targeted inhibitory receptors and alternative immune checkpoint molecules. Tumours under selective pressure from PD-1 or CTLA-4 blockade may elevate expression of TIM-3, LAG-3, or VISTA, reducing the efficacy of monoclonal antibody therapy and restoring local immunosuppression [169]. Changes in the cytokine milieu driven by both tumour and stromal cells can further promote immune cell exhaustion and dysfunction, diminishing long-term immunotherapeutic benefit [170].

### Microenvironment-mediated and systemic resistance

Tumour-extrinsic mechanisms that influence both primary and acquired resistance. TME and systemic factors strongly influence both inherent and acquired resistance. Hypoxic conditions, metabolic competition for nutrients such as glucose, and accumulation of immunosuppressive metabolites (e.g., adenosine, lactic acid) can blunt T cell activity and antigen presentation. Additionally, emerging research highlights the impact of the gut microbiome on systemic immune tone, with certain microbial compositions correlating with improved response or resistance to ICIs [171–173].

In summary, resistance to immunotherapy arises from a tightly interwoven set of tumour-intrinsic genetic alterations, dynamic extrinsic microenvironmental factors, and adaptive immune responses. The identification and integration of predictive biomarkers ranging from TMB and neoantigen burden to microenvironmental signatures are pivotal for improved patient stratification and the rational design of next-generation combination therapies [172]. Continued mechanistic studies will drive advances in overcoming resistance, expanding the reach of immunotherapy to broader patient populations and a wider spectrum of cancers.

The multifactorial nature of immune checkpoint inhibitor resistance is summarized in Table 4, which categorizes primary, acquired, and microenvironment-mediated mechanisms alongside key molecular pathways, representative biomarkers, and emerging therapeutic strategies.

**Table 4:** Mechanisms of cancer cell resistance to immune checkpoint inhibitors (ICIs)

Mechanism	Sub-mechanism	Key molecular/cellular features	Representative references
Primary (Intrinsic) Resistance	Tumor Antigenicity and Antigen Presentation	• Low tumor mutational burden (TMB) or paucity of immunogenic neoantigens limits generation of high-affinity T-cell clones, reducing tumor recognition. • Defects in antigen-processing machinery, including mutations or loss of β2-microglobulin (B2M) or MHC-I heavy chain, impair presentation of peptide–MHC complexes to CD8⁺ T cells.	[143, 144]
	Immune-Excluded (“Cold”) Tumor Microenvironment	• Poor infiltration of tumor-infiltrating lymphocytes (TILs) and low interferon signatures characterize immune-desert phenotypes. • Overexpression of immunosuppressive cytokines (e.g., TGF-β, IL-10) and altered chemokine gradients create physical/functional barriers to effector cell recruitment.	[145, 146]
	Oncogenic Pathway Activation	• Aberrant WNT/β-catenin signaling suppresses CCL4 and dendritic cell recruitment. • PTEN loss/PI3K activation and MAPK pathway mutations down-regulate interferon responses, driving T-cell exclusion.	[149, 150]
2. Acquired (Secondary) Resistance	Antigen Loss or Editing	Immune pressure selects for antigen-loss variants through down-regulation or mutation of target epitopes, rendering tumor cells invisible to reinvigorated T cells.	[150]
	Interferon-γ Pathway Disruption	Mutations in JAK1/2, STAT1, or IRF1 disrupt IFN-γ signaling, reducing MHC expression and preventing effector-mediated killing.	[149]
	Up-regulation of Alternative Checkpoints	Adaptive increases in TIM-3, LAG-3, VISTA, TIGIT, or BTLA re-establish inhibitory signaling despite PD-1/CTLA-4 blockade.	[151]
	Cytokine & Metabolic Adaptations	Chronic therapy promotes an immunosuppressive milieu enriched in IL-8, VEGF, and adenosine, driving T-cell exhaustion and expansion of regulatory cell populations.	[152]
3. Microenvironment-Mediated and Systemic Resistance	–	• Metabolic competition: Hypoxia, glucose depletion, and accumulation of lactate or adenosine impair T-cell glycolysis and cytokine production. • Immunosuppressive cell populations: Regulatory T cells (Tregs), myeloid-derived suppressor cells (MDSCs), and tumor-associated macrophages (TAMs) secrete TGF-β, IL-10, and express checkpoint ligands. • Gut microbiome composition: Enrichment of Akkermansia muciniphila or Bifidobacterium correlates with improved ICI responsiveness, whereas dysbiosis associates with resistance.	[147, 148, 153-155]
4. Biomarker Development & Therapeutic Implications	–	• Predictive biomarkers: PD-L1 immunohistochemistry (TPS/CPS), TMB, gene-expression signatures, and multiplexed spatial profiling are under active validation for patient stratification. • Combination strategies: Targeting parallel inhibitory receptors (e.g., PD-1 + LAG-3 blockade), restoring antigen presentation (e.g., epigenetic modulators), and metabolic reprogramming (e.g., adenosine antagonists) are being explored to overcome resistance.	[141, 142]

## Future challenges and directions in cancer immunotherapy

### Refining predictive biomarkers for immunotherapy response

Despite the transformative clinical impact of immune checkpoint inhibitors and other immunotherapeutic modalities, important challenges remain in optimizing their application and extending benefits to a broader patient population [174]. A major focus of ongoing research lies in identifying and validating robust predictive biomarkers that can reliably reliably determine which malignancies and patient subsets are most likely to derive the greatest benefit from immunotherapy [175].

Current biomarkers including tumour mutational burden (TMB), microsatellite instability (MSI), mismatch repair (MMR) deficiency, and PD-L1 expression—have been instrumental in patient selection but lack universal predictive value across cancer types and treatment modalities. This limitation underscores the need for a new generation of dynamic and multidimensional biomarkers capable of capturing the complexity of the tumour–immune interface.

### Multi-omics approaches in biomarker discovery

Multi-omics–driven biomarker discovery is therefore emerging as a critical frontier. Integrative profiling platforms that combine whole-exome sequencing (WES) for neoantigen prediction, single-cell RNA sequencing (scRNA-seq) for immune landscape deconvolution, and spatial transcriptomics such as 10× Genomics Visium, NanoString GeoMx for mapping cell–cell interactions within the tumour microenvironment (TME) now enable unprecedented resolution of tumour–immune crosstalk.

In parallel, serum proteomics (Olink or SOMAscan) and circulating tumour DNA (ctDNA) analysis through ultra-deep hybrid capture sequencing are being leveraged to develop dynamic, blood-based biomarkers for real-time monitoring of treatment response and minimal residual disease [176, 177]. These platforms provide not only static molecular portraits but also temporal insights into treatment-induced immune remodeling, facilitating adaptive therapeutic decisions and early detection of immune escape. Recent studies illustrate these advances: a meta-analysis in urothelial carcinoma demonstrated that baseline ctDNA detectability and on-treatment decline correlate with improved PFS and OS [178, 179]. Similarly, Integrating Bulk, Single-cell, and Spatial Transcriptomics analyses in NSCLC have yielded prognostic signatures, such as the Combined Cell Death Index, that stratify response to ICIs [180].

### Computational integration and machine learning pipelines

Beyond data generation, progress increasingly depends on computational integration of multi-omics pipelines. Machine learning frameworks including multi-modal variational autoencoders (MVAEs) and graph-based are being applied to unify genomic, epigenomic, transcriptomic, and proteomic datasets [181, 182].

Such computational tools can identify convergent immune-evasive signatures that are not apparent in single-omic analyses, thereby guiding patient stratification and informing rational drug combinations. For example, a recent preprint introduced a “Biologically Disentangled Variational Autoencoder (BDVAE)” that integrates transcriptomic and genomic data and achieves AUC-ROC ≈0.94 for predicting ICI response in a pan-cancer cohort, while uncovering mechanisms of immune suppression, metabolic shifts, and neuronal signaling associated with resistance.

### Rational combination strategies to overcome resistance

The clinical integration of combination modalities remains a major frontier in overcoming both primary and acquired resistance. Ongoing preclinical and clinical studies are actively evaluating rational combinations of immunotherapeutic agents with established treatment modalities, including tyrosine kinase inhibitors (TKIs), conventional cytotoxic chemotherapies, and radiotherapy [183]. TKIs, by modulating angiogenic factors and immune-suppressive cell populations within the TME, may augment immunotherapeutic efficacy through synergistic mechanisms [184]. Similarly, chemotherapy and radiotherapy, long regarded as immunosuppressive, are now known to induce immunogenic cell death and potentiate antigen release, thereby amplifying anti-tumour immune responses when used in concert with immune checkpoint blockade [185]. These synergistic effects form the basis for rational combination strategies aimed at enhancing immune activation and overcoming resistance to ICI therapy. Figure 2 illustrates the key mechanisms underlying ICI activity and highlights how adjunct treatments such as chemotherapy and radiotherapy can enhance therapeutic efficacy.

### Emerging platforms: synthetic biology and engineered immunotherapies

Beyond classical agents, novel immune modulators and engineered cellular platforms are entering early-phase trials. These include bispecific checkpoint inhibitors, oncolytic viruses armed with immune stimulatory transgenes, and next-generation CAR-T/NK cells engineered with PD-1 dominant negative receptors or inducible cytokine circuits [186]. Synthetic biology approaches are enabling the programmable control of cytokine release or checkpoint receptor expression, while computational trial-design tools are facilitating the development of adaptive clinical protocols that dynamically incorporate multi-omic biomarkers to guide patient enrolment and treatment escalation. For example, a recent review by Zhu et al. [187] describes synthetic biology strategies to improve specificity and controllability of engineered immune cells and gene circuits in cancer immunotherapy, including the development of intratumoral disease-sensing circuits and engineered cytokine payloads that mitigate systemic toxicity [188]. Also, the MANIFEST study (2025) is an observational multi-omic platform in which transcriptomic, proteomic, and immune cell profiling are used prospectively to predict and monitor response in patients receiving ICIs, illustrating how omics biomarkers are being incorporated into real-world patient selection and follow-up [189].

### Adaptive trial designs and precision dosing

Prospective trials are increasingly leveraging adaptive designs to refine dosing, scheduling, and sequencing of immunotherapeutic regimens. The I-SPY2.2 SMART trial in breast cancer utilizes a Bayesian adaptive randomization framework to dynamically reassign non-responders to alternative immunotherapy arms based on interim response data. Similarly, the Precision Dose-Finding (PDF) design integrates pharmacokinetic (PK) modeling to individualize dosing in Phase I oncology trials, optimizing efficacy while minimizing toxicity.

These innovations exemplify the transition toward real-time, data-informed clinical decision-making, reinforcing the symbiosis between clinical design and computational analytics.

### Expanding horizons: adoptive cell therapies, vaccines, and oncolytic viruses

Careful patient selection remains critical for the successful implementation of combination and next-generation regimens. Adaptive biomarker-guided trials, such as the Rapid Integral Biomarker-Adapted Feasibility Study, now use IHC-based stratification to tailor combination immunotherapies [190].

Emerging therapeutic strategies continue to explore the potential of novel immunomodulators, adoptive cell therapies, oncolytic viruses, and cancer vaccines, both as monotherapies and within combinatorial frameworks. Advances such as epigenetic modulation such as the enhancer of zeste homolog 1/2 (EZH1/EZH2) inhibition have recently been shown to enhance adoptive T cell immunotherapy in multiple cancer models [191]. Synthetic biology and advances in computational modeling are anticipated to revolutionize the design and customization of immunotherapeutic regimens tailored to individual patient and tumour characteristics.

For example, Lifileucel (AMTAGVI), a tumour-infiltrating lymphocyte (TIL) therapy approved in 2024, is being combined with ICIs in advanced melanoma to deepen and prolong responses [192]. Recent reviews (e.g., Peng et al. 2025) have highlighted new vaccine platforms—including peptide, mRNA, and viral-vector vaccines—that enhance neoantigen priming and immune infiltration in “cold” tumours [193]. In parallel, precision-engineered oncolytic viruses such as CLD-201 (vaccinia-based, stem cell-loaded) and genetically optimized OVs for colorectal cancer are redefining viral immunotherapy by improving specificity and immune activation [194].

### AI-driven modeling and personalized immunotherapy design

Artificial intelligence (AI) and computational modeling are accelerating personalized immunotherapy design. A recent AI-driven hybrid ecological model for oncolytic viral therapy (OVT) integrated time-delayed Lotka–Volterra equations with reinforcement learning to predict treatment dynamics and identify biomarker signatures (TNF-α, IL-18, NF-κB) associated with therapeutic efficacy [195].

These predictive tools, combined with engineered cellular and viral vectors carrying inducible immune circuits, are expected to enable self-regulating immunotherapies tailored to individual tumour biology and immune contexture.

In conclusion, the future of cancer immunotherapy will depend on a deeper mechanistic understanding of tumour–immune interactions, the integration of multi-omic biomarkers, and computationally informed adaptive clinical design. Progress in these domains will rely on multidisciplinary collaboration, real-world validation, and the seamless convergence of experimental biology with digital analytics.

Collectively, these advances promise to transform immunotherapy from a population-level intervention into a truly personalized, dynamically optimized treatment paradigm capable of delivering durable remission across diverse malignancies.

## Conclusion

The history and evolution of immunotherapy in cancer management vividly illustrate the remarkable achievements borne from decades of scientific inquiry and translational research. Initial forays into immunotherapeutic strategies were limited by an incomplete understanding of tumour immunology; however, progressive elucidation of cancer biology particularly the complexities of the TME and the dynamic interplay between neoplastic cells and cytotoxic T lymphocytes has been pivotal in advancing this therapeutic field.

The characterization of immune checkpoints as critical modulators of T-cell activation and function has incontrovertibly transformed oncologic paradigms. The successive development and clinical approval of ICIs, including those targeting CTLA-4, PD-1, and PD-L1, have redefined treatment algorithms across a diverse spectrum of malignancies and disease stages. These agents, acting through selective blockade of immune-inhibitory pathways, have conferred durable clinical benefit and, in select cases, produced unprecedented long-term survivorship in historically intractable cancers.

Moreover, the progressive integration of immunotherapy into both first line and subsequent therapeutic settings across various tumour types underscore the versatility and adaptability of immune-based interventions. These developments reflect not only innovation in drug discovery but also an evolving understanding of patient selection, biomarker-driven therapy, and the molecular determinants of treatment response.

Despite these substantial breakthroughs, the phenomenon of resistance both primary and acquired remains a formidable barrier to universal success. Recent insights into the mechanistic underpinnings of immune escape, including alterations within the TME, loss of antigenicity, and modulation of immune inhibitory circuits, have provided critical avenues for ongoing research and therapeutic innovation. The recognition of distinct resistance mechanisms is instrumental in informing rational combination strategies, novel therapeutic targets, and personalized approaches to overcoming therapeutic failure.

Collectively, the trajectory of immunotherapy from experimental modality to established standard-of-care mirrors the broader progress in cancer biology and translational oncology. Continued advances in our mechanistic understanding of tumour-immune dynamics will enable the next generation of immunotherapeutics, with the promise of further improving outcomes and expanding the benefits of immunotherapy to a broader patient population.

In summation, the reviewed evidence not only highlights the historical milestones and evolving landscape of immunotherapy but also accentuates the imperative of ongoing scientific inquiry to overcome the adaptive resilience of cancer. As we move forward, an integrated approach encompassing molecular insights, innovative therapeutic strategies, and rigorous clinical evaluation will be central to realizing the full potential of immunotherapeutic intervention in oncology.

## Data Availability

No datasets were generated or analysed during the current study.

## Abbreviations

APC: Antigen-presenting cell
BCC.: Basal cell carcinoma
cSCC.: Cutaneous squamous cell carcinoma
CTLA-4: Cytotoxic T-lymphocyte antigen 4
ECM: Extracellular matrix
GEJ.: Gastroesophageal junction
HMC: Histocompatibility complex
HNSCC: Head and neck squamous cell carcinoma
HCC: Hepatocellular carcinoma
ICI: Immune checkpoint inhibitor
IL-10: Interleukin-10
MCC.: Merkel cell carcinoma
MSI-H: Microsatellite instability-high
dMMR: Mismatch repair deficiency
MDSCs.: Myeloid-derived suppressor cells
NSCLC: Non-small cell lung cancer
PD1: Programmed cell death protein 1
PD-L1: Programmed cell death protein ligand 1
PD-L2: Programmed cell death protein ligand 2
RCC: Renal cell carcinoma
SCLC: Small cell lung cancer
TCR: T cell receptors
TGF-β: Transforming growth factor-beta
T-regs: T-regulatory cells
TNBC.: Triple-negative breast cancer
TME: Tumour microenvironment
TILs: Tumour-infiltrating lymphocytes
TMB: Tumour mutation burden
TAMs: Tumour-associated macrophages
TKI: Tyrosine kinase inhibitor
irAEs: Immune-related adverse events
EZH1/EZH2: Enhancer of zeste homolog 1/2
Bat3: HLA-B-associated transcript 3
Fyn/TYK2: Fyn tyrosine kinase/tyrosine kinase 2
MHC II: Major histocompatibility complex class II
LSECtin: Sinusoidal endothelial cell lectin
PLCγ1: Phospholipase C gamma 1
PKC: Protein kinase C
NF-κB: Nuclear factor kappa B
JAK/STAT: Janus kinase/signal transducer and activator of transcription
BATF: Basic Leucine Zipper ATF-Like transcription factor
T-bet: T-box expressed in T cells
c-Myc: Cellular myelocytomatosis oncogene
mTORC1/2: Mammalian target of rapamycin complex ½
CD3ζ: Cluster of differentiation 3 zeta chain
ZAP-70: Zeta-chain- associated protein kinase 70
MAPK: Mitogen-activated protein kinase
IL-10: Interleukin-10
TGF-β: Transforming growth factor beta
MDSCs: Myeloid-derived suppressor cells
MYC: Myelocytomatosis oncogene
PTEN: Phosphatase and Tensin homolog
TMB: Tumour mutational burden
